# Maternal near-miss surveillance, Namibia

**DOI:** 10.2471/BLT.20.251371

**Published:** 2020-07-01

**Authors:** Steffie Heemelaar, Mirjam Josef, Zoe Diener, Melody Chipeio, Jelle Stekelenburg, Thomas van den Akker, Shonag Mackenzie

**Affiliations:** a Department of Obstetrics & Gynaecology, Katutura State Hospital, PO Box 86237, Eros, Windhoek, Namibia.; b Department of Obstetrics & Gynaecology, University of North Carolina, North Carolina, United States of America.; c Department of Health Science, Global Health, University Medical Center Groningen, Groningen, Netherlands.; d Department of Obstetrics, Leiden University Medical Center, Leiden, Netherlands.; e Department of Obstetrics & Gynaecology, University of Namibia, Windhoek, Namibia.; Correspondence to Steffie Heemelaar (email: s.heemelaar@lumc.nl).

## Abstract

**Objective:**

To analyse and improve the Namibian maternity care system by implementing maternal near-miss surveillance during 1 October 2018 and 31 March 2019, and identifying the challenges and benefits of such data collection.

**Methods:**

From the results of an initial feasibility study, we adapted the World Health Organization’s criteria defining a maternal near miss to the Namibian health-care system. We visited most (27 out of 35) participating facilities before implementation and provided training on maternal near-miss identification and data collection. We visited all facilities at the end of the surveillance period to verify recorded data and to give staff the opportunity to provide feedback.

**Findings:**

During the 6-month period, we recorded 37 106 live births, 298 maternal near misses (8.0 per 1000 live births) and 23 maternal deaths (62.0 per 100 000 live births). We observed that obstetric haemorrhage and hypertensive disorders were the most common causes of maternal near misses (each 92/298; 30.9%). Of the 49 maternal near misses due to pregnancies with abortive outcomes, ectopic pregnancy was the most common cause (36/298; 12.1%). Fetal or neonatal outcomes were poor; only 50.3% (157/312) of the infants born to maternal near-miss mothers went home with their mother.

**Conclusion:**

Maternal near-miss surveillance is a useful intervention to identify within-country challenges, such as lack of access to caesarean section or hysterectomy. Knowledge of these challenges can be used by policy-makers and programme managers in the development of locally tailored targeted interventions to improve maternal outcome in their setting.

## Résumé

**Objectif:**

Analyser et améliorer le système de soins obstétricaux namibien en instaurant une surveillance des décès maternels évités de justesse durant la période comprise entre le 1^er^ octobre 2018 et le 31 mars 2019. Cerner les défis et avantages que représente une telle collecte de données.

**Méthodes:**

En nous fondant sur les résultats d'une étude de faisabilité initiale, nous avons adapté les critères employés par l'Organisation mondiale de la Santé pour définir les décès maternels évités de justesse et les avons appliqués au système de santé namibien. Nous sommes allés dans la plupart (27 sur 35) des établissements participants avant la mise en œuvre, et avons organisé des formations consacrées à l'identification des décès maternels évités de justesse et à la collecte de données. Nous avons également rendu visite à l'ensemble des établissements à l'issue de la période de surveillance afin de vérifier les données recueillies et de donner au personnel soignant l'occasion d'exprimer son avis.

**Résultats:**

Durant cette période de 6 mois, nous avons enregistré 37 106 naissances vivantes, 298 décès maternels évités de justesse (8,0 pour 1000 naissances vivantes) et 23 décès maternels (62,0 pour 100 000 naissances vivantes). Nous avons constaté que les causes les plus fréquentes de décès maternels évités de justesse étaient les hémorragies obstétricales et les problèmes d'hypertension (92/298 pour l'une comme pour l'autre; 30,9%). Sur les 49 décès maternels évités de justesse liés à des grossesses infructueuses, la cause la plus répandue était la grossesse extra-utérine (36/298; 12,1%). Le nombre d'issues fœtales ou néonatales était faible; à peine 50,3% (157/312) des bébés nés de femmes ayant évité un décès de justesse sont rentrés avec leur mère à la sortie de l'hôpital.

**Conclusion:**

La surveillance des décès maternels évités de justesse est une intervention utile pour identifier les défis auxquels est confronté le pays, comme le manque d'accès aux césariennes ou aux hystérectomies. S'ils sont conscients de ces défis, les législateurs et responsables de programmes peuvent développer des actions ciblées et adaptées aux spécificités locales afin d'améliorer la santé maternelle dans leur région.

## Resumen

**Objetivo:**

Analizar y mejorar el sistema de atención de maternidad de Namibia al implementar la vigilancia de la morbilidad materna extrema durante el 1 de octubre de 2018 y el 31 de marzo de 2019, e identificar los retos y los beneficios de esa recopilación de datos.

**Métodos:**

A partir de los resultados de un estudio inicial de viabilidad, se adaptaron los criterios de la Organización Mundial de la Salud que definen la morbilidad materna extrema al sistema de atención sanitaria de Namibia. Se visitaron la mayoría (27 de 35) de los centros participantes antes de la implementación y se impartió capacitación sobre la identificación de los casos de morbilidad materna extrema y la recopilación de datos. Se visitaron todos los centros al final del periodo de vigilancia para verificar los datos registrados y dar al personal la oportunidad de proporcionar información.

**Resultados:**

Durante el periodo de 6 meses, se registraron 37 106 nacimientos vivos, 298 casos de morbilidad materna extrema (8,0 por cada 1000 nacimientos vivos) y 23 muertes maternas (62,0 por cada 100 000 nacimientos vivos). Se observó que las hemorragias obstétricas y los trastornos hipertensivos fueron las causas más comunes de las morbilidades maternas extremas (cada una de ellas 92/298; 30,9 %). De los 49 casos de morbilidad materna extrema por embarazos cuyo resultado fue un aborto, el embarazo ectópico fue la causa más común (36/298; 12,1 %). Los resultados fetales o neonatales fueron deficientes; solo el 50,3 % (157/312) de los infantes nacidos de madres con morbilidad materna extrema regresaron a casa junto con sus madres quienes habían sido dadas de alta.

**Conclusión:**

La vigilancia de la morbilidad materna extrema es una intervención de gran utilidad para identificar las dificultades que existen dentro del país, como la falta de acceso a la intervención de cesárea o la histerectomía. Los responsables de formular las políticas y los gestores de los programas pueden utilizar el conocimiento de estas dificultades en la elaboración de iniciativas focalizadas y adaptadas a las necesidades locales para mejorar los resultados de la maternidad en su entorno.

## ملخص

الغرض تحليل وتحسين نظام رعاية الأمومة في ناميبيا عن طريق تنفيذ مراقبة حالات نجاة الأمهات من الوفاة المؤكدة، خلال الفترة من 1 أكتوبر/تشرين أول 2018 إلى 31 مارس/آذار 2019، وتحديد التحديات والفوائد تجميع هذه البيانات.

الطريقة من نتائج دراسة الجدوى الأولية، قمنا بتكييف معايير منظمة الصحة العالمية التي تُعرّف حالة نجاة الأمهات من الوفاة شبه المؤكدة، في نظام الرعاية الصحية الناميبي. قمنا بزيارة معظم المنشآت المشاركة (27 من إجمالي 35) قبل التنفيذ، وقدمنا التدريب على تحديد حالات نجاة الأمهات من الموت شبه المؤكدة، وجمع البيانات. وقمنا بزيارة جميع المنشآت في نهاية فترة المراقبة للتحقق من البيانات المسجلة، ولإعطاء فريق العمل فرصة تقديم الملاحظات.

النتائج خلال فترة 6 أشهر، قمنا بتسجيل 37106 حالة ولادة حية، و298 حالة نجاة للأمهات من الوفاة المؤكدة (8.0 لكل 1000 حالة ولادة حية)، و23 حالة وفاة للأمهات (62.0 لكل 100000 حالة ولادة حية). ولاحظنا أن النزيف أثناء الولادة، واضطرابات فرط ضغط الدم، كانت أكثر الأسباب شيوعاً لحالات قرب الأمهات من الوفاة (كل 92/298؛ 30.9%). من بين 49 حالة نجاة لأمهات من الوفاة شبه المؤكدة بسبب الحمل مع نتائج للإجهاض، كان الحمل خارج الرحم هو السبب الأكثر شيوعاً (36/298؛ 12.1%). كانت النتائج المتعلقة بالجنين أو الأطفال حديثي الولادة بسيطة؛ فقط 50.3% (157/312) من المواليد وُلدوا لأمهات كانت على وشك الوفاة المؤكدة، وعادوا إلى المنزل مع أمهاتهم بعد الولادة.

الاستنتاج تعد مراقبة حالات نجاة الأمهات من الوفاة شبه المؤكدة، تدخلاً مفيداً لتحديد التحديات داخل البلد، مثل عدم توفر فرصة العملية القيصرية أو استئصال الرحم. إن معرفة هذه التحديات يمكن استغلالها من جانب صانعي السياسات ومديري البرامج في تطوير التدخلات المستهدفة المصممة محلياً، لتحسين نتائج الولادة في بيئاتهم.

## 摘要

**目标:**

旨在通过于 2018 年 10 月 1 日和 2019 年 3 月 31 日期间实施危重孕产妇监测来分析和改善纳米比亚孕产妇护理系统，并确定收集此类数据的挑战和益处。

**方法:**

根据初步可行性研究的结果，我们调整了世界卫生组织定义危重孕产妇的标准，使之适用于纳米比亚的医疗保健系统。在实施之前，我们访问了大部分参与机构（35 家中的 27 家），并对其进行有关危重孕产妇的识别和数据收集培训。我们在监测期结束时访问了所有机构以核实记录的数据，并为工作人员提供分享反馈的机会。

**结果:**

在这 6 个月期间，我们记录了 37,106 例活产儿，298 例危重孕产妇（每 1000 例活产儿中有 8.0 例）和 23 例孕产妇死亡（每 100000 例活产儿中有 62.0 例）。我们发现，产科出血和高血压疾病是导致危重孕产妇最常见的原因（均为 92/298；30.9%）。在 49 例最终导致流产的危重孕产妇中，宫外孕是最常见的原因（36/298；12.1%）。胎儿或新生儿生命体征弱；危重孕产妇生下的婴儿中，只有 50.3% (157/312) 的婴儿可以和母亲一起出院回家。

**结论:**

危重孕产妇监测是一种有效识别国内挑战的干预手段，例如缺乏实施剖腹产或子宫切除术的机会。政策制定者和计划管理者可以利用对这些挑战的了解来针对当地状况制定干预措施，以改善纳米比亚医疗背景下的孕产妇结果。

## Резюме

**Цель:**

Анализ и совершенствование действующей системы охраны материнства в Намибии посредством мониторинга осложнений при родах, представляющих угрозу для жизни, в период с 1 октября 2018 г. по 31 марта 2019 г., а также выявление проблем и преимуществ такого сбора данных.

**Методы:**

Ориентируясь на результаты предварительного анализа осуществимости, авторы адаптировали критерии Всемирной организации здравоохранения, определяющие понятие осложнений при родах, представляющих угрозу для жизни, к реалиям системы здравоохранения в Намибии. До начала мониторинга авторы посетили большую часть (27 из 35) учреждений, участвовавших в исследовании, и провели обучение по выявлению осложнений при родах, представляющих угрозу для жизни, и сбору данных. По окончании периода мониторинга авторы посетили все учреждения, чтобы проверить точность зарегистрированных данных и предоставить персоналу возможность высказать свое мнение.

**Результаты:**

На протяжении 6 месяцев наблюдений авторами было зарегистрировано 37 106 живорожденных, 298 случаев осложнений при родах, представлявших угрозу для жизни (8,0 на 1000 живорожденных), и 23 случая материнской смерти (62,0 случая на 100 000 живорожденных). Согласно наблюдениям, кровотечения в родах и гипертензивные расстройства были наиболее распространенными причинами осложнений при родах, представляющих угрозу для жизни (каждая из причин составляла 92 из 298 случаев, 30,9%). В 49 случаях осложнений, представлявших угрозу для жизни, в результате беременности, завершившейся выкидышем, наиболее распространенной причиной была внематочная беременность (36 случаев из 298, 12,1%). Показатели здоровья плода или новорожденного были низкими: только 50,3% (157 из 312) младенцев, рожденных матерями, имевшими осложнения при родах, представлявшими угрозу для жизни, были выписаны вместе с матерями.

**Вывод:**

Мониторинг осложнений во время родов, представляющих угрозу для жизни, является полезным вмешательством, позволяющим выявить проблемы внутри страны, такие как недостаточный доступ к кесареву сечению или гистерэктомии. Понимание этих проблем может помочь руководителям программ и лицам, принимающим решения, в разработке специализированных программ вмешательств на местах для улучшения состояния здоровья матерей в соответствующих условиях.

## Introduction

A target within the third sustainable development goal (SDG 3: ensure healthy lives and promote well-being for all at all ages)^1^ is to reduce the maternal mortality ratio to 70 per 100 000 live births globally by 2030. Even though the global maternal mortality ratio was reduced by nearly half in 2015 compared with 1990, large discrepancies remain between regions; the highest maternal mortality ratio of 546 was recorded in sub-Saharan Africa, compared with 12 deaths per 100 000 live births in high-income countries.^2^ Namibia, a middle-income country in sub-Saharan Africa, is one of the least densely populated countries in the world (2.8 people per km^2^) and has around 70 000 births per annum.^3^^,^^4^ Although Namibia had an estimated maternal mortality ratio of 385 per 100 000 live births in 2013, the government is committed to achieving SDG 3.^5^^,^^6^

To analyse and improve the maternity care system, the World Health Organization (WHO) recommends including maternal near misses, that is, severe maternal morbidity, defined by a specific set of criteria, within national obstetric surveillance systems.^7^ National surveillance of maternal near misses, or other specifically defined maternal morbidities, is conducted in several high-income countries, but such data collection occurs infrequently in low- or middle-income countries.^8^^,^^9^

The low absolute numbers of maternal deaths in Namibia present a challenge to the monitoring of the performance of the maternity care system. After an initial feasibility study, the National Maternal Death Review Committee of the Ministry of Health and Social Services agreed to include surveillance of maternal near misses within the national obstetric surveillance system for a 6-month period. The aim of this surveillance was to obtain more robust data on pregnancy outcomes and assess the benefits of such surveillance in comparison with maternal death surveillance only.

We describe the implementation of maternal near-miss surveillance from 1 October 2018 to 31 March 2019 in Namibia, and discuss the challenges and benefits of such data collection. Using a cross-sectional study design, we provide nationwide incidence data and discuss the underlying causes of maternal near misses, while also examining neonatal outcomes.

## Methods

### Study setting

All Namibian public hospitals, 1 tertiary, 4 regional and 30 district, participated in the surveillance of maternal near misses. The largest hospital complex, located in the capital of Windhoek and comprising the tertiary and a regional hospital, has around 12 000 births per annum. This hospital employs three consultant obstetrician-gynaecologists. The intensive care unit has advanced equipment including ventilators and dialysis. The other three regional hospitals (6500 births per year each) have high-dependency units with mechanical ventilation, and renal dialysis can be performed at two of these hospitals. District hospitals have two to eight general medical doctors who provide care across all specialities. District hospitals have basic haematology and chemistry laboratory tests available, such as blood count, renal function and basic liver function tests. Most district hospital blood banks only have access to 2 units of packed red cells. All hospitals are expected to have functioning operating theatres for basic surgical procedures, such as caesarean section or laparotomy for ruptured ectopic pregnancy.

### Feasibility study and preparation

As a result of the limited availability of laboratory tests and management options resulting in the underreporting of maternal near-miss cases, other sub-Saharan African countries have indicated that the WHO maternal near-miss criteria may not be suitable for use in district hospitals in low-income settings.^10^^,^^11^ As 30 of the 35 Namibian hospitals are district hospitals, four of the authors of this study, together with several other clinicians working in Namibian public facilities, conducted a feasibility study in 2018 in four hospitals to compare WHO maternal near-miss criteria with a set of criteria proposed for low-income settings.^12^ This study was performed in the hospital complex in the capital and in a regional and two district hospitals. The authors of the feasibility study reported that the WHO criteria resulted in the underreporting of maternal near misses in Namibia; we therefore adapted the WHO maternal near-miss identification criteria to the Namibian health-care system (Box 1).^13^ Within management-based criteria, we adopted the lower threshold of 4 units of blood transfused and included laparotomy; we also included eclampsia and uterine rupture within the category of severe maternal complications.

Box 1 **Maternal near-miss criteria as defined by the World Health Organization and as locally amended for Namibia**

**Clinical criteria**

WHO: Acute cyanosis; gasping; respiratory rate > 40 or < 6/min; shock;^a^ oliguria non-responsive to fluids or diuretics;^b^ failure to form clots; loss of consciousness lasting > 12 hours (Glasgow coma scale < 10); cardiac arrest; stroke; uncontrollable fit/total paralysis; and jaundice in the presence of pre-eclampsia.

Namibia: the same as WHO.

**Laboratory-based criteria**

WHO: Oxygen saturation < 90% for 60 minutes; *Pa*o_2_/FiO2 < 200 mmHg; creatinine 300 μmol/L or 3.5 mg/dL; bilirubin > 100 mmol/L or > 6.0 mg/dL; pH < 7.1; lactate > 5 mq/mL; acute thrombocytopenia (< 50 000 platelets/mL); and loss of consciousness and ketoacids in urine.

Namibia: the same as WHO.

**Management-based criteria**

WHO: Use of continuous vasoactive drugs; hysterectomy following infection or haemorrhage; transfusion of 5 units of red blood cells; intubation and ventilation for 60 minutes not related to anaesthesia; dialysis for acute renal failure; cardio–pulmonary resuscitation.

Namibia: As for WHO with the exception of transfusion of 4 units of blood products, and inclusion of laparotomy other than caesarean section or ectopic pregnancy of < 12 weeks gestation

**Severe maternal complications**

WHO: No criteria.

Namibia: Eclampsia and uterine rupture.^c^

FiO_2_: fraction of inspired oxygen; min: minute(s); *Pa*o_2_: arterial oxygen partial pressure; WHO: World Health Organization. ^a^ Persistent systolic blood pressure of < 80 mmHg, or a persistent systolic blood pressure < 90 mmHg with a pulse rate of ≥ 120 bpm. ^b^ Urinary output < 30 mL/hour over 4 hours or < 400 mL per 24 hours. ^c^ Complete rupture of uterus during labour confirmed by laparotomy.

Before national implementation of maternal near-miss surveillance, most participating facilities were visited; due to a lack of funding, eight smaller district hospitals could not be visited. Medical staff involved in the care of pregnant and/or postpartum women were trained in the identification of maternal near misses and relevant data collection. Staff at the eight hospitals that could not be visited received training when presenting at one of the referral hospitals (either a regional hospital or a larger, better-equipped district hospital) for other training courses. At all 35 hospitals, a maternal near-miss doctor and nurse were nominated to supervise data collection and provide the research team with verbal monthly updates during pre-arranged telephone calls.

### Data collection

A case of a maternal near miss was defined as a woman either pregnant (independent of gestational age), or within 42 days of termination of pregnancy or birth, fulfilling at least one of the criteria listed in Box 1. Using a structured data collection tool (Maternal Near Miss Form, available in the data repository),^14^ nominated staff collected anonymous data from medical records on maternal sociodemographic characteristics, maternal outcome, the main underlying cause of the maternal near miss and the neonatal outcome. Stillbirths were defined as deaths before birth after 28 weeks of gestation, and documented as either fresh or macerated in the medical file. Neonatal death was defined as the death of an infant within the first 28 days of life. Since we aimed to assess maternal outcome, neonatal outcome was assessed upon discharge of the mother even if the infant was still being cared for in the intensive care unit.

We identified possible missed cases in the Windhoek hospital complex during weekly ward visits or through personal communication with nominated medical staff. Although we had planned to visit all facilities 2 months after the onset of the surveillance, we had to cancel these visits because of a lack of resources. At the request of staff at two of the hospitals, we made an extra visit to provide additional training on data collection. After 6 months, we visited all hospitals to verify the recorded surveillance data against medical records. We screened the ward registers of maternity, female, high-dependency and intensive care units, theatre registers and referral registers for missed cases. During this visit, at least one member of the National Maternal Death Review Committee met with the local hospital staff, including the nominated doctor and nurse, the doctor and nurse in-charge, and all available doctors and nurses involved in the care of pregnant women. During these meetings, staff were given the opportunity to describe their experience with data collection and the challenges encountered related to clinical duties.

We obtained the total number of live births in Namibia from the National Health Information Systems. We collected data on maternal deaths from the national reporting and audit system.^15^

### Data analysis

Direct and indirect causes of maternal near misses were defined according to the International Statistical Classification of Diseases-Maternal Mortality definitions.^16^ We defined the number of severe maternal outcomes as the total of maternal near misses and maternal deaths. We calculated the incidence of the most common causes of, and other conditions contributing to, maternal near misses, namely major obstetric haemorrhage, eclampsia, uterine rupture and hysterectomy, per 1000 live births during the study period. We defined mortality index as the number of maternal deaths as a percentage of the number of severe maternal outcomes. 

As a result of poor documentation of blood loss, we diagnosed major obstetric haemorrhage as a woman with obstetric haemorrhage who either needed 4 units of blood; fulfilled the criteria of shock;^17^ had a laparotomy (to perform a B-lynch) or a hysterectomy; or had disseminated intravascular coagulopathy, requiring fresh frozen plasma. To diagnose eclampsia, uterine rupture and hysterectomy, we used definitions proposed by the International Network of Obstetric Survey Systems.^18^

Finally, because an outbreak of hepatitis E had been causing significant maternal mortality since December 2017, we also calculated the incidence and mortality index of severe maternal outcomes for women with acute hepatitis E with a bilirubin concentration of more than 100 mmol/L.^19^

### Dissemination of findings

We shared all findings with the executive management committee of the Ministry of Health and Social Services during a meeting in July 2019, attended by representatives of the departments of human resources, clinical support services and quality assurance division, responsible for training clinical staff. We addressed several issues and set priorities for the following year, namely: (i) the human resources department to focus on recruiting and retaining doctors and nurses with obstetric experience and/or essential surgical skills; and (ii) the clinical support services department to ensure functionality of operating theatres in district hospitals. We also discussed the possibility of launching a debate within parliament to legalize abortion, using data describing abortion-related complications. 

In the same month we also shared all findings with all participating facilities through video conferencing and during a 2-day national conference organized by the Ministry of Health and Social Services and the University of Namibia, funded by several Namibian companies, the WHO, United Nations Population Funds and the European Union. A doctor and nurse from each facility were invited to attend the conference, where we provided staff training and issued relevant guidelines according to the most common issues identified in the maternal near miss and death reviews. The guidelines were written by doctors working in maternity departments of the regional hospitals, and reviewed by members of the National Maternal Death Review Committee. 

### Budget

Costs were kept as low as possible. When feasible, facility visits for maternal near-miss surveillance were combined with visits for other training courses. Most of the available budget was spent on travel and accommodation; the costs for two committee members to travel 9600 km to visit participating hospitals before and after completion of data collection were approximately 8000 United States dollars (US$). A further US$ 700 was spent on stationary, such as the printing of case reporting forms and guidelines. External advisors from outside Namibia were not remunerated.

### Ethics

This study was reviewed and approved by the research unit of the Ministry of Health and Social Services. After stabilizing and treating the women, data were collected from medical records without identification of the patient; inclusion in the study had no effect on clinical management. The need for individual informed consent was therefore waived.

## Results

Over the 6-month surveillance period, we recorded 37 106 live births, 298 maternal near misses and 23 maternal deaths. We calculated the incidence of maternal near misses in Namibia as 8.0 per 1000 live births, the maternal mortality ratio as 62.0 per 100 000 live births and the ratio of maternal near misses to maternal deaths as 13:1.

We list the characteristics of the women identified as having experienced a maternal near miss or death in Table 1. Among the women who experienced a severe maternal outcome, 18.1% (58/321) were between the ages of 13 and 19 years and 30.8% (99/321) were primiparous.

**Table 1:** Characteristics of all women who experienced a maternal near miss or maternal death, Namibia, 1 October 2018–31 March 2019

Characteristics	No. (%)		
	Maternal near misses (*n* = 298)	Maternal deaths (*n* = 23)	Severe maternal outcomes (*n* = 321)
**Age (years)**			
≤ 20	54 (18.1)	4 (17.4)	58 (18.1)
21–34	174 (58.4)	15 (65.2)	189 (58.9)
≥ 35	69 (23.2)	4 (17.4)	73 (22.7)
Unknown	1 (0.3)	0 (0.0)	1 (0.3)
**Parity**			
0	96 (32.2)	3 (13.0)	99 (30.8)
1–3	149 (50.0)	15 (65.2)	164 (51.1)
4	38 (12.8)	5 (21.7)	43 (13.4)
Unknown	15 (5.0)	0 (0.0)	15 (4.7)
**Antenatal care attendance**			
Yes	199 (66.8)	21 (91.3)	220 (68.5)
No	20 (6.7)	2 (8.7)	22 (6.9)
NA^a^	51 (17.1)	0 (0.0)	51 (15.9)
Unknown	28 (9.4)	0 (0.0)	28 (8.7)
**Gestational age (weeks)**			
≤ 12	36 (12.1)	0 (0.0)	36 (11.2)
13–25	20 (6.7)	4 (17.4)	24 (7.5)
26–36	104 (34.9)	9 (39.1)	113 (35.2)
≥ 37	108 (36.2)	9 (39.1)	117 (36.4)
Unknown	30 (10.1)	1 (4.3)	31 (9.7)
**Previous caesarean section**			
Yes	66 (22.1)	6 (26.1)	72 (22.4)
No	212 (71.1)	17 (73.9)	229 (71.3)
Unknown	20 (6.7)	0 (0.0)	20 (6.2)
**HIV status**			
Positive	36 (12.1)	6 (26.1)	42 (13.1)
Negative	222 (74.5)	15 (65.2)	237 (73.8)
Unknown	40 (13.4)	2 (8.7)	42 (13.1)
**Pregnancy outcome**			
Normal vaginal birth	73 (24.5)	11 (47.8)	84 (26.2)
Instrumental birth	2 (0.7)	1 (4.3)	3 (0.9)
Caesarean section	137 (46.0)	5 (21.7)	142 (44.2)
Laparotomy uterine rupture	15 (5.0)	0 (0.0)	15 (4.7)
Miscarriage	15 (5.0)	1 (4.3)	16 (5.0)
Ectopic	38 (12.8)	0 (0.0)	38 (11.8)
Still pregnant at discharge	15 (5.0)	5 (21.7)	20 (6.2)
Termination of pregnancy	2 (0.7)	0 (0.0)	2 (0.6)
Unknown	1 (0.3)	0 (0.0)	1 (0.3)

HIV: human immunodeficiency virus; NA: not applicable.

^a^ Gestation < 20 weeks.

**Fig. 1:**
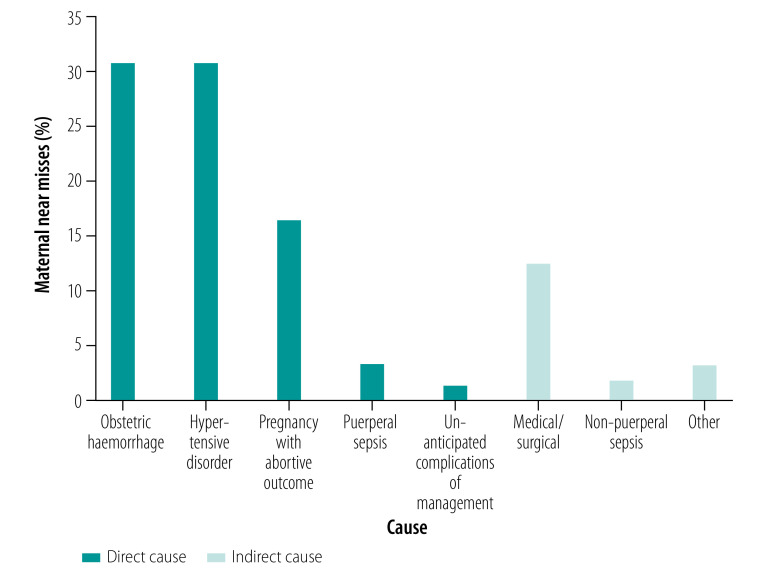
Direct and indirect causes of maternal near misses, Namibia, 1 October 2018–31 March 2019

The main underlying causes of maternal near misses are summarized in Fig. 1 and reported in detail in Table 2. We observed that the most common causes were obstetric haemorrhage (92/298; 30.9%) and hypertensive disorders (92/298; 30.9%). Of the 49 maternal near misses, due to pregnancies with abortive outcomes, ectopic pregnancy was the most common underlying cause (36/298; 12.1%). Ten women experienced a septic miscarriage, recorded in the medical file to be the result of self-induced abortion in five women. Of these self-induced abortions, two were complicated by a ruptured uterus and one of these needed a hysterectomy. One woman had a perforated uterus after self-induced abortion using a branch.

**Table 2:** Direct and indirect causes of maternal near misses, Namibia, 1 October 2018–31 March 2019

Cause of maternal near miss (*n* = 298)	No. of maternal near misses (%)
**Direct cause**	
Obstetric haemorrhage	92 (30.9)
Postpartum haemorrhage	34 (11.4)
Abruptio placentae	27 (9.1)
Uterine rupture	15 (5.0)
Placenta praevia	7 (2.3)
Placenta accreta^a^	5 (1.7)
Bleeding related to caesarean section^b^	12 (4.0)
Hypertensive disorder	92 (30.9)
Eclampsia^c^	71 (23.8)
HELLP syndrome	20 (6.7)
Pre-eclampsia	3 (1.0)
Pulmonary oedema	5 (1.7)
Stroke	1 (0.3)
Pregnancy with abortive outcome	49 (16.4)
Ectopic	36 (12.1)
Septic miscarriage^d^	10 (3.4)
Abortion-related haemorrhage	1 (0.3)
Ruptured uterus	1 (0.3)
Heterotopic pregnancy	1 (0.3)
Puerperal sepsis	10 (3.4)
Post emergency caesarean section	5 (1.7)
Post elective caesarean section	3 (1.0)
Post vaginal delivery	2 (0.7)
Unanticipated complications of management	4 (1.3)
Aspiration pneumonia	1 (0.3)
Laryngospasm post intubation	1 (0.3)
Massive intra-abdominal haematoma post caesarean section	1 (0.3)
Pubic diastasis post normal vaginal birth, needing open reduction and internal fixation	1 (0.3)
**Indirect cause**	
Medical or surgical	37 (12.4)
Hepatitis E	23 (7.7)
Cardiac disorder	5 (1.7)
Acute abdomen needing laparotomy	4 (1.3)
Pancytopaenia	1 (0.3)
Epilepsy	1 (0.3)
Guillain-Barré Syndrome	1 (0.3)
Bowel gangrene post herbal intoxication	1 (0.3)
Stab wound	1 (0.3)
Non-puerperal sepsis	5 (1.7)
Liver abscess	1 (0.3)
Tuberculosis	1 (0.3)
Pneumonia	1 (0.3)
Pelvic inflammatory disease, grade IV	1 (0.3)
Meningitis	1 (0.3)
Other	9 (3.0)
Chronic anaemia	5 (1.7)
Pulmonary oedema, cause unclear	2 (0.7)
Domestic violence	1 (0.3)
Morbidly obese	1 (0.3)

HELLP: haemolysis, elevated liver enzymes and low platelet count.

^a^ Two of these women also had placenta praevia.

^b^ Five of these women also had postpartum haemorrhage and one had placental abruption.

^c^ Eight of these women also had HELLP syndrome.

^d^ Two of these women also had a ruptured uterus after self-induced abortion, and one after induction with misoprostol for missed miscarriage. One woman arrived in septic shock with a perforated uterus after self-induced abortion using a branch.

Direct causes of maternal deaths were haemorrhage (three), hypertension (two), puerperal sepsis after caesarean section (two) and anaesthesia-related complications (two). Indirect causes were hepatitis E (five), cardiac disorder (four), tuberculosis (two) and gastric perforation (one). The cause of death remained unclear for two women.

We present incidence calculations and mortality indices in Table 3. We observed the highest incidence of a severe maternal outcome for massive obstetric haemorrhage (2.3 per 1000 live births). We calculated the highest mortality index for women with hepatitis E (5/28; 17.9%).

**Table 3:** Number of maternal near misses and deaths plus incidence and mortality index for the most common direct and indirect causes, Namibia, 1 October 2018–31 March 2019

Variable	Massive obstetric haemorrhage	Eclampsia	Uterine rupture	Hysterectomy	Hepatitis E, bilirubin > 100 mmol/L
No. maternal near misses (*n* = 298)	83	73	18	23	23
No. maternal deaths (*n* = 23)	3	1	1	1	5
Incidence of severe maternal outcomes per 1000 live births^a^	2.3	2.0	0.5	0.6	0.8
Mortality index (%)^b^	3.5	1.4	5.3	4.2	17.9

^a^ Total number of live births during the study period was 37 106.

^b^ Number of maternal deaths as a percentage of number of severe maternal outcomes (i.e. number of maternal near misses plus number maternal deaths).

The proportion of births by caesarean section varied across the regions from 2.7% (114/4203) in Ohangwena to 30.5% (1033/3392) in Oshana (Table 4). For five women in need of a caesarean section, their uterus ruptured on the way from district to referral hospital. A hysterectomy was performed in 24 women (0.6 per 1000 live births), of which five were as a result of sepsis and 19 as a result of haemorrhage; 45.8% (11/24) of these women had received at least one caesarean section previously. A B-lynch suture to prevent haemorrhage-related hysterectomy was placed in only one of the 19 women. For six other women who experienced a massive obstetric haemorrhage, a B-lynch suture was placed and no hysterectomy was needed.

**Table 4:** Regional birth numbers and types of deliveries, Namibia, 1 October 2018–31 March 2019

Region	No. of live births	No. (%) of normal vaginal births	No. (%) of assisted vaginal births	No. (%) of caesarean sections	Total no. of births
Erongo	2 451	2 086 (84.1)	1 (0.0)	393 (15.8)	2 480
Hardap	1 140	924 (83.9)	3 (0.3)	174 (15.8)	1 101
Karas	1 085	901 (82.4)	11 (1.0)	182 (16.6)	1 094
Kavango (east and west)	4 052	3 585 (86.6)	35 (0.8)	520 (12.6)	4 140
Khomas	6 211	4 777 (78.4)	49 (0.8)	1 269 (20.8)	6 095
Kunene	1 327	1 276 (94.9)	0 (0.0)	69 (5.1)	1 345
Ohangwena	4 147	4 086 (97.2)	3 (0.1)	114 (2.7)	4 203
Omaheke	1 206	1 051 (86.8)	3 (0.2)	157 (13.0)	1 211
Omusati	3 820	3 640 (94.3)	18 (0.5)	200 (5.2)	3 858
Oshana	3 386	2 357 (69.5)	2 (0.1)	1 033 (30.5)	3 392
Oshikoto	4 285	3 599 (84.0)	0 (0.0)	687 (16.0)	4 286
Otjozondjupa	2 353	2 129 (89.0)	6 (0.3)	258 (10.8)	2 393
Zambezi	1 643	1 527 (94.6)	0 (0.0)	87 (5.4)	1 614
**Total**	**37 106**	**31 938 (85.8)**	**131 (0.4)**	**5 143 (13.8)**	**37 212**

Fetal and neonatal outcomes were poor (Table 5). Only 50.3% (157/312) of the infants went home alive at the time of discharge of the mother. Two pregnancies were terminated before the fetus had reached a viable gestational age: one woman had early-onset severe pre-eclampsia at 21 weeks gestation and one woman had an acute abdomen caused by bowel strangulation at 22 weeks gestation.

**Table 5:** Fetal or neonatal outcome of the 298 maternal near misses, Namibia, 1 October 2018–31 March 2019

Fetal or neonatal outcome (*n* = 312)^a^	No. (%)
Alive upon discharge of mother	157 (50.3)
Admitted to neonatal intensive care unit	33 (10.6)
Fresh stillborn	33 (10.6)
Macerated stillborn	6 (1.9)
Neonatal death	5 (1.6)
Terminated pregnancy	2 (0.6)
Miscarriage/ectopic	54 (17.3)
Mother still pregnant at discharge	15 (4.8)
Unknown	7 (2.2)

^a^ There were 14 twin pregnancies.

The most common challenge reported by medical personnel was understaffing and lack of experience in the staff present. Basic equipment, including blood pressure machines, was often lacking or not functioning. In 13 of the 30 district hospitals, no or only a few uncomplicated caesarean sections or laparotomies for ectopic pregnancies were performed as a result of a lack of skills, staff or equipment.

## Discussion

Our collection of maternal near-miss data, in addition to routine maternal death analysis, has proven to be useful for several reasons. First, these data provide insights into the functioning of the health-care system, and several issues were addressed immediately. With less than 1500 live births per annum, maternal deaths seldom occur in district hospitals. The challenges met by these smaller facilities, such as a lack of access to basic surgery, have been highlighted. For several women, this lack of access to surgery led to a maternal near-miss complication, such as uterine rupture or shock during transport to a referral hospital. We observed a low prevalence (0.6 per 1000 live births) of the life-saving intervention of hysterectomy, one of the few management options for haemorrhage when medical treatment fails; the relevant surgical skills are only available in eight of Namibia’s 35 hospitals. This prevalence is lower than the global estimate of 0.9 per 1000 live births or 1.4 per 1000 live births in a South African district.^20^^,^^21^ Underreporting is not likely to be the cause of this low prevalence, as missed cases were easily identified through theatre registers.

Second, an unanticipated, but important benefit of maternal near-miss data collection was the positive effect on the morale of staff. Medical personnel are working under difficult circumstances with a high workload and constrained resources. However, by participating in the maternal near-miss surveillance, staff could take pride in their jobs by acknowledging the number of women they had saved.

Third, as maternal near misses are more frequent than maternal deaths, near-miss data may be more useful in advocacy, for instance in abortion-related complications. Using data on the extent and complications of unsafe abortions was key in the battle to legalize abortion in Rwanda.^22^

Our study had several limitations. First, we only aimed to describe the implementation of maternal near-miss surveillance, although it is the resulting improvement in maternal outcome that is important. As surveillance continues, we anticipate that an apparent increase in the incidence of near misses will be observed as a result of improved data collection, rather than any change in maternal outcome. Similarly, an apparent increase in maternal deaths was reported in South Africa as a result of the implementation of national maternal death reviews.^23^ Any observed trends may also be affected by the simultaneous implementation of other quality-improving projects, such as the provision of maternal death review feedback from a national to facility level, and the provision of emergency obstetric care courses to clinical staff and final-year medical students.

Second, our calculated incidence of maternal near misses must be interpreted with caution, as we amended criteria to the local situation. Although this approach may hamper international comparison, the use of WHO criteria led to severe underreporting of maternal near misses in the feasibility study conducted previously because of the limited diagnostics and management options in smaller facilities.^13^ Two other middle-income countries, Brazil and Nigeria, implemented national maternal near-miss surveillance while adhering strictly to WHO identification criteria.^24^^,^^25^ However, only tertiary health facilities participated in the Brazil and Nigeria surveys, in which the applicability of WHO criteria may not have been problematic, as such facilities are generally better equipped than smaller hospitals.

Third, our calculated maternal mortality ratio (62.0 per 100 000 live births) is much lower than that estimated by the Demographic Health Survey or WHO.^3^^,^^6^ On analysis of the annual maternal death review findings, the National Maternal Death Review Committee assumed this result is due to underreporting; the fact that the maternal mortality ratio varied widely between regions is most likely explained by differences in quality of reporting rather than differences in quality of care. Similar or even larger discrepancies between WHO estimates and national maternal death reviews were also found in Ethiopia, Malawi and South Africa.^26^^–^^28^ Such large discrepancies need to be explored further, as progress in improving quality of care can only be monitored with a reliable maternal mortality ratio.

Fourth, our biggest challenge to successful implementation of the surveillance was a limited budget. We were not able to fund any administrative staff to support data collection or visit every participating facility before the onset of the surveillance. Cases may have been missed, affecting the quality of our data. However, as data collection depended on local staff, the approach gave them the opportunity to show leadership and take ownership of data collection. In general, it appeared staff were more likely to collect accurate data when trained personally and when aware of the aim of the project. Barriers to accurate data collection by local staff were lack of time due to a high workload and the fear of making mistakes in data collection.

The maternal near-miss surveillance identified several issues that need to be explored further before they can be addressed. First, over 80% of maternal near misses were the result of direct causes compared with less than half of the maternal deaths, which corresponds with findings in Brazil and Nigeria.^24^^,^^29^^–^^33^ This difference could be the result of high mortality indexes among several indirect causes, such as observed for hepatitis E and described for some cardiac disorders, human immunodeficiency virus and tuberculosis, common among young women in Namibia.^32^^,^^34^^–^^36^ However, underreporting of maternal near misses as a result of indirect causes is also likely; such patients were more likely to have been on general wards, where maternal near-miss data collection was not being supervised.

Second, nearly half of the women who experienced a maternal near miss during our 6-month survey gave birth by caesarean section; caesarean sections are used for varying proportions of births across the regions, by up to as many of 30% of births at a population level (Table 4). The highest proportions are partly explained by the fact that some regions (namely Karas, Khomas, Oshana and Oshikoto) contain referral and regional hospitals; however, overuse of caesarean section, despite the well-known complications of this procedure, is also likely.^37^ Over one-fifth of our study population had experienced a caesarean section previously and among the women who needed a hysterectomy this proportion was almost half. Importantly, an instrumental vaginal birth, which could potentially prevent birth by caesarean section, was rarely performed in both our maternal near-miss and the general pregnant population: a common finding in low- and middle-income settings.^38^^,^^39^ The inadequate use of these two potential lifesaving interventions (the overuse of caesarean section and underuse of instrumental vaginal birth), a problem in many countries,^40^ and its potential negative effect on maternal outcome needs to be urgently assessed in Namibia.

Third, overall fetal and neonatal outcomes were poor, which is commonly seen among women with severe morbidity.^41^ To assess the role of quality of care, neonatal outcome must be assessed in correlation with maternal condition.^29^^,^^32^ Poor neonatal outcome due to uterine rupture is seen even in high-income countries, although neonatal outcome is better after a spontaneous vaginal birth complicated by postpartum haemorrhage.^42^

We identified two potentially important facilitators that will support the continuation of maternal near-miss surveillance in Namibia: its independence of the availability of donor funding and the motivation of staff to continue collecting data. Causes of maternal death are similar for most countries, but individual countries experience local challenges. Maternal near-miss surveillance is a useful intervention to identify these challenges, the knowledge of which can be used by policy-makers and programme managers in the development of locally tailored targeted interventions to improve maternal outcome in their setting.
